# The Physiological Responses of Wheat and Maize Seedlings Grown under Water Deficit Are Modulated by Pre-Application of Auxin-Type Plant Growth Regulators

**DOI:** 10.3390/plants11233251

**Published:** 2022-11-26

**Authors:** Dessislava Todorova, Zornitsa Katerova, Elena Shopova, Liliana Brankova, Iskren Sergiev, Jurga Jankauskienė, Sigita Jurkonienė

**Affiliations:** 1 Institute of Plant Physiology and Genetics, Bulgarian Academy of Sciences, Acad. G. Bonchev Str., Bl. 21, 1113 Sofia, Bulgaria; 2 Nature Research Centre, Laboratory of Plant Physiology, Institute of Botany, 2 Akademijos Str., 08412 Vilnius, Lithuania

**Keywords:** antioxidants, auxin-type compounds, PEG-6000, *Triticum aestivum* (L.), *Zea mays* (L.)

## Abstract

The physiological responses of wheat and maize seedlings to exogenous auxin-type compounds 1-[2-chloroethoxycarbonyl-methyl]-4-naphthalenesulfonic acid calcium salt (TA-12) and 1-[2-dimethylaminoethoxicarbonylmethyl]naphthalene chlormethylate (TA-14) application prior to polyethyleneglycol-6000 (PEG) treatment were studied. PEG treatment inhibited seedlings growth and caused alterations in their antioxidant defence which was crop-specific. PEG increased the non-enzymatic antioxidants along with inhibition of enzymatic antioxidant activity in wheat, while in maize the opposite effects were found. The TA-12 and TA-14 applied alone increased most of the growth parameters measured in both crops, as well as the catalase activity and protein content of wheat. The growth of PEG-treated wheat and maize plants was improved by foliar spray with TA-compounds (TAs). Application of TAs before PEG treatment maintained low-molecular weight thiol-containing compounds and protein contents, and catalase and peroxidase activities close to the control levels. This was better expressed in maize than in wheat seedlings. The results showed that the preliminary application of TA-12 and TA-14 can reduce the adverse effects of moderate water deficit by crop-specific adjustment of the antioxidant defence to counteract stress.

## 1. Introduction

Drought negatively impacts agricultural production on a worldwide scale and is one of the most widespread abiotic stress factors due to global warming and the sequential climate changes. It might be induced by water deficiency, by high temperatures and/or evaporation, and by a combination of these factors [1,2]. Drought affects all aspects of plant physiology, including a reduction of photosynthesis rate and overproduction of reactive oxygen species (ROS), resulting in retarded plant growth and significant crop losses [2,3]. Plants have developed antioxidant defence system that consists of enzymatic (superoxide dismutase, SOD; catalase, CAT; guaiacol peroxidase, POX, etc.) and non-enzymatic (ascorbate, glutathione, phenolics, proline, etc.) components [3,4], which change significantly in response to different stresses, including drought. 

The positive physiological defense responses induced by different compounds and causing adaptive cellular response to subsequent exposure to stressor is known as chemical priming [5,6]. The induced resistance to a given stressor after priming could be manifested by induction of various phytohormone-signaling pathways, secondary metabolites, defense proteins, elevation of ROS as signaling molecules, activation of antioxidant defense systems, etc. Alagna et al. [5] emphasized the necessity to expand the knowledge of the factors responsible for plant resistance and plant tolerance obtained due to priming on both physiological and molecular levels, especially necessary for monocotyledons.

Auxins control various aspects of plant growth and development, including plant response to environmental stressors. Together with other phytohormones, they interact with ROS and participate in the signaling and drought-response of plants [7,8,9]. For example, exogenous IAA was shown to induce ABA and JA contents, to activate drought stress responsive genes and to reduce the negative physiological effects of drought on white clover [10].

The synthetic auxins 1-[2-chloroethoxycarbonyl-methyl]-4-naphthalenesulfonic acid calcium salt (TA-12) and 1-[2-dimethylaminoethoxicarbonylmethyl] naphthalenechlormethylate (TA-14) used in the current study are structural analogues of naphthyl acetic acid. They were previously analyzed as prospected protectors against different abiotic stress factors—cold stress in rapeseed [11,12]; UV-B and UV-C irradiation [13,14]; high temperature [15]; drought stress [16]; and as modulators of herbicidal stress [17,18] in pea plants. 

Wheat, rice, and maize are ranked as the three most important staple crops [19,20,21]. Based on meta-analyses of the existing literature records, Daryanto et al. [20] reported that maize had higher sensitivity to drought than wheat. By process-based crop models, Webber et al. [22] predicted that drought will cause higher yield losses for maize than for wheat. Recently, Ostrowska and Hura [23] selected quick photosynthetical parameters for the evaluation of the reactions to water stress (including drought) of maize and wheat which are possessing C4 and C3 type of photosynthesis, respectively. 

The potential of the auxin-type plant growth regulators (TA-12 and TA-14) as modulators of the physiological response to drought stress in wheat and maize has not been explored until now. Wheat and maize were selected for the current study because it is known that they differ in their drought tolerance, being relatively tolerant and very sensitive to drought, respectively [20]. The study aims to examine the physiological response of young wheat and maize seedlings to moderate water deficit, after foliar application with TA-12 and TA-14. It expands the knowledge on the physiological effects of the synthetic auxins on two economically important crop plants grown under water deficit stress conditions.

## 2. Results

### 2.1. Growth Parameters: Length and Fresh Weight of Shoots and Roots

**Figure 1:**
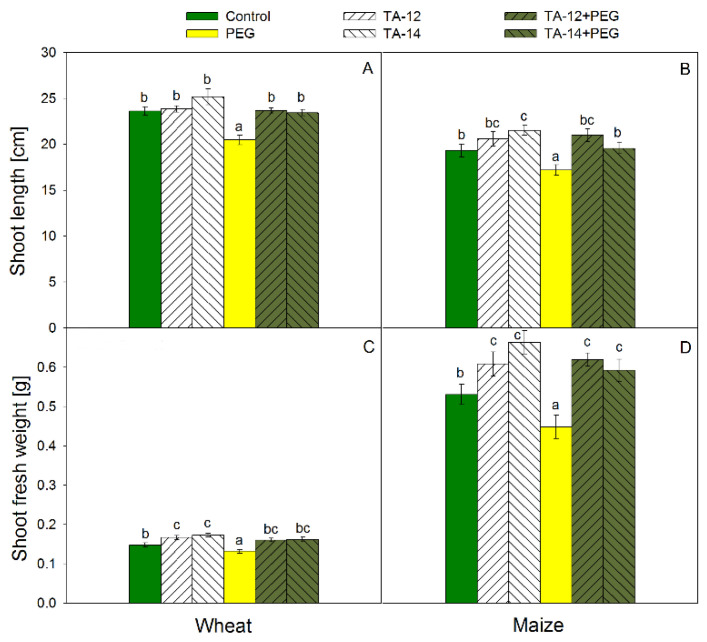
Shoot length of wheat (**A**) and maize (**B**) and shoot fresh weight of wheat (**C**) and maize (**D**) foliar sprayed with 1 mM TA-12 or TA-14 and subjected to 10% PEG for 72 h. Data are mean values ± SE. Different letters designate statistically significant difference at *p* < 0.05.

**Figure 2:**
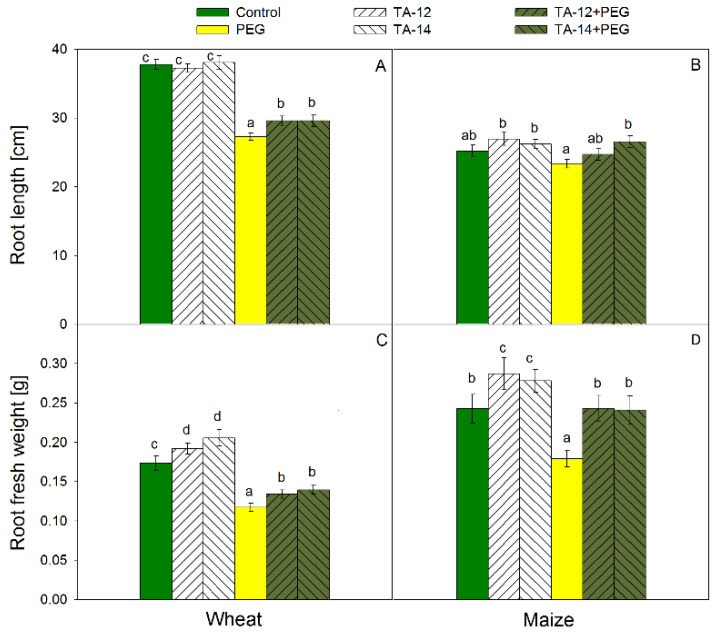
Root length of wheat (**A**) and maize (**B**) and root fresh weight of wheat (**C**) and maize (**D**) foliar sprayed with 1 mM TA-12 or TA-14 and subjected to 10% PEG for 72 h. Data are mean values ± SE. Different letters designate statistically significant difference at *p* < 0.05.

The alterations of wheat and maize length and fresh weight after PEG treatment and TAs application are presented in Figure 1 (for shoots) and Figure 2 (for roots). The length of wheat (Figure 1A) and maize (Figure 1B) shoots decreased significantly due to PEG treatment. The application of TA-12 + PEG and TA-14 + PEG restored the length of wheat and maize shoots back to the control levels. Applied alone, TA-14 increased the length of maize shoots only. Both TA-12 and TA-14 applied alone raised fresh weight of wheat (Figure 1C) and maize (Figure 1D) shoots but a reverse tendency was found after PEG treatment. The combined application of TA-12 + PEG and TA-14 + PEG restored the fresh weight of wheat shoots to the control levels (Figure 1C), while the fresh weight of maize shoots was increased above the control (Figure 1D). 

Root length decreased significantly in wheat (Figure 2A) and not considerably in maize (Figure 2B) due to PEG treatment. The length of wheat root was increased due to combined application of TA-12 + PEG and TA-14 + PEG as compared with PEG treatment, but remained lower than the control levels. Within treatments, the alterations of maize root length varied less as compared to wheat (Figure 2B): a significant reduction was found only after PEG treatment as compared to the alone application of TAs. The root fresh weight of wheat (Figure 2C) and maize (Figure 2D) increased significantly due to the alone application of TAs but decreased substantially after PEG treatment. The combined application of TA-12 + PEG and TA-14 + PEG increased root fresh weight of wheat as compared with PEG treatment, but remained significantly lower than the control values (Figure 2C). On the contrary, the combined application of TA-12 + PEG and TA-14 + PEG restored entirely the fresh weight of maize roots back to the control levels (Figure 2D). 

### 2.2. Content of Free Proline, Total Phenolics and Free Low-Molecular Weight Thiols

**Figure 3:**
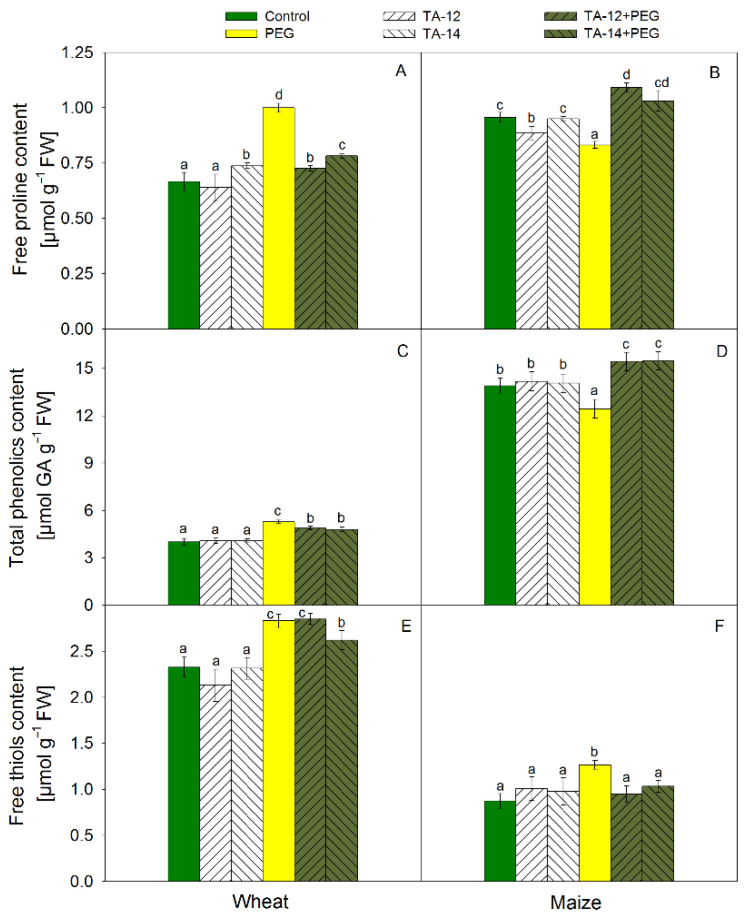
Free proline (**A**,**B**), total phenolics (**C**,**D**), and free low-molecular weight thiols (**E**,**F**) contents in shoots of wheat (**A**,**C**,**E**) and maize (**B**,**D**,**F**) foliar sprayed with 1 mM TA-12 or TA-14 and subjected to 10% PEG for 72 h. Data are mean values ± SE. Different letters designate a statistically significant difference at *p* < 0.05.

The contents of free proline, total phenolics, and free low-molecular weight thiols are shown on Figure 3. Slight induction of free proline was observed after alone application of TA-14 in wheat (Figure 3A) but a significant reduction was detected due to TA-12 in maize (Figure 3B). PEG treatment caused a substantial increase of free proline in wheat while the opposite trend was found in maize. After the combined application of TA-12 + PEG and TA-14 + PEG, proline content decreased significantly in wheat as compared to PEG treatment, but remained higher than the control levels (Figure 3A). In maize, its content decreased considerably due to PEG treatment, but increased after combined application of TA + PEG (Figure 3B).

The content of total phenolics in wheat increased after PEG and the combined application of TA-12 + PEG and TA-14 + PEG (Figure 3C). The values of wheat total phenolics due to combined application become closer to the control value than those, obtained after PEG treatment. The content of maize total phenolics decreased due to PEG treatment and increased after the combined application of TA-12 + PEG and TA-14 + PEG (Figure 3D).

The low-molecular weight free thiols (−SH) content increased in wheat after PEG and the TA-12 + PEG and TA-14 + PEG application (Figure 3E). Application of TA-14 + PEG led to small reduction of –SH content as compared to PEG treatment. The −SH content increased in maize after PEG treatment (Figure 3F) while TA-12 + PEG and TA-14 + PEG application restored control values.

### 2.3. Activity of Antioxidant Enzymes

**Figure 4:**
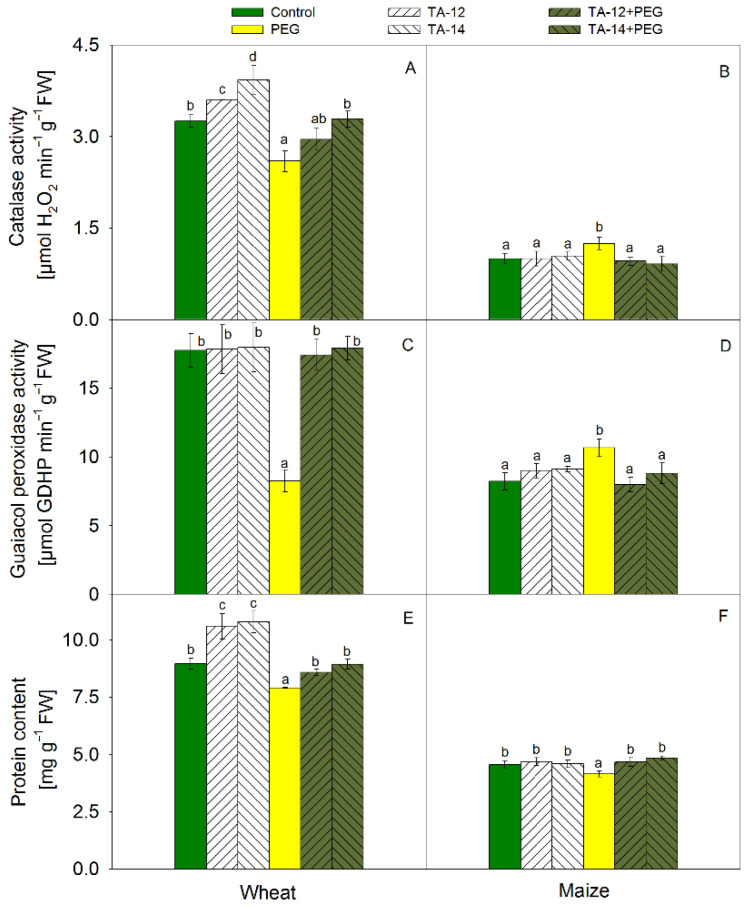
Activities of catalase (CAT; **A**,**B**) and guaiacol peroxidase (**C**,**D**), and protein content (**E**,**F**) in shoots of wheat (**A**,**C**,**E**) and maize (**B**,**D**,**F**) foliar sprayed with 1 mM TA-12 or TA-14 and subjected to 10% PEG for 72 h. Data are mean values ± SE. Different letters designate a statistically significant difference at *p* < 0.05.

Increased CAT activity was detected in wheat due to TAs application (Figure 4A). On the contrary, a significant reduction of CAT activity was found in PEG-treated wheat. In contrast to wheat, CAT activity was slightly increased in PEG-treated maize (Figure 4B) but the combined application of TA-12 + PEG and TA-14 + PEG restored the control values in both crops. 

The activity of POX decreased significantly in PEG-treated wheat (Figure 4C) but increased in maize (Figure 4D). Application of TA + PEG restored POX activity to the control values in both plants.

Increased protein content was detected in wheat after TAs application but PEG treatment led to its reduction (Figure 4E). Protein content was decreased slightly in PEG-treated maize (Figure 4F). Pretreatment with TAs restored protein content in PEG-treated seedlings.

## 3. Discussion

Drought might be induced by the limitation of water availability to the roots or by excessive transpiration from leaves. Insufficient equilibrium between water uptake and transpiration lead to the development of osmotic stress, in parallel with oxidative stress [2]. The supply of PEG in hydroponics is frequently used to imitate artificial drought stress conditions in laboratory experiments [2]. In distinct of low-molecular weight osmolytes used to impose osmotic stress, the high molecular weight (≥6000) PEG is not able to penetrate the cell wall. If roots are damaged, for instance by aeration of hydroponics, then PEG can enter the tissue and along with osmotic stress, PEG could provoke toxic contamination [24]. Another problem using PEG to impose drought stress is related to its ability to create boundary layers that could cause hypoxic conditions to roots, especially when there is no additional aeration [24]. This is valid in solutions with water potentials lower than −0.8 MPa (severe drought stress) because the high viscosity of PEG limits the diffusion of O_2_. Consequently, the probability of root O_2_ deficiency to occur is increasing. Therefore, in our study we applied PEG-6000 to simulate artificial moderate (−0.2 MPa) drought stress but without aeration in order to avoid root wounding, which would cause an additional PEG toxicity stress.

The drought responses of plants can be influenced by phytohormones, including auxins [8,9]. The plant hormones regulate many physiological processes in plants and recently the need to understand the interplay between ROS and phytohormones during stress was emphasized [8]. Yadav et al. [9] inferred that indol-3-il acetic acid is implicated in drought stress governance through activation of other stress responsive phytohormones as well as the generation of ROS, which can act as signal molecules. Here, we explore for the first time, the physiological effect of the preliminary application of auxin-type plant growth regulators (PGRs), TA-12 and TA-14, prior to PEG treatment in the staple crops wheat and maize. The emphasis is on assessment of the capabilities of TA-12 and TA-14 to modulate the consequences of PEG-induced drought in young seedlings. The obtained information will shed more light on the action of auxin-type PGRs on the effects of water deficit.

The slight induction of the fresh weight after application of TAs is not surprising as auxin is known to be involved in various cellular and plant growth processes [25]. In a previous study on *Pisum sativum* L., TA-12 and TA-14 were found to increase the growth of pea [13,14,18]. PEG causes growth inhibition as it was already reported [16,26,27]. The preliminary application of TAs counteracted the negative effect of PEG treatment on shoot and root length of maize but only partially in shoots of wheat that seems to be due to a species-specific differences. Similarly, TAs increased fresh weight of maize near to the control (roots) and even higher levels (shoots). The beneficial effect of TAs on wheat root growth was minor. It could be speculated that foliar applied auxin-type PGR during its transport to the roots might be metabolized in different ways in both crops, which could explain the weaker effect on wheat root growth.

Proline has a multiple role in plant stress reactions such as osmoprotective compatible solute; ROS scavenger; energy supplying compound during stress recovery or stress bioindicator; etc. [28]. According to Bhaskara et al. [29], proline turnover, rather than only its accumulation, has the main role in growth support under drought stress. The connection of proline and redox status is emphasized to be of great importance for plant performance under water stress [29,30]. Increased proline content was observed after drought stress by many authors in different plants: wheat [26,31,32,33], *Carex duriuscula* [34], *Hordeum vulgare* L. [35], *Brassica juncea* L. [36], etc., including maize [37]. Our results for proline content in wheat plantlets subjected to PEG are in agreement with previous publications. The information regarding phytohormonal contribution in regulation of proline metabolism under drought is scarce [30]. Sadiqov et al. [31] showed that pre-application of indole-3-il acetic acid decreased proline accumulation in wheat grown under drought stress. Therefore the observed reduction of proline content after TAs + PEG treatment of wheat plants could be due to the auxin nature of the applied compounds. Accumulation of proline or other compatible solutes might be connected with the improvement of drought tolerance [38]. It was reported that after moderate or severe drought, proline content increased to a different extent in maize hybrids depending on their tolerance [38]. The accumulation of proline under water deficit conditions could contribute to plant’s drought tolerance or could be a stress-injury indicator depending on plant species and severity of the stress [39]. Our study showed that the growth inhibition due to PEG application in maize was concomitant with the reduction of proline content, which is in line with the concept that maize is very sensitive to drought, especially in the seedling stage [21]. The induction of proline accumulation due to the combined application of TAs + PEG in maize plantlets seemed to modulate positively the antioxidant status (CAT and POX activity) and plant growth performance under drought. Our result corresponds well with the generally accepted idea that proline accumulation had high contribution in drought tolerance [30].

It is well known that drought stress, similarly to other abiotic stressors induces deleterious amounts of different ROS compounds and initiates membrane damages in plant cells [2,3]. Phenolics are part of the low-molecular antioxidant defense system implicated as an ROS scavenger [3,40]. In general, drought stress led to the induction of phenolic compounds [33,38,41,42], which is in line with our data observed for wheat. The opposite results found for maize could be related to species specific differences. The maintenance of phenolics values near to the control due to the combined application of TAs + PEG in both wheat and maize could indicate that the preliminary application of auxin-type PGRs may partially adjust ROS homeostasis in young plants. Our data support the already promoted hypothesis that auxin could regulate ROS homeostasis [8,16,43].

Low-molecular weight thiols, containing mainly glutathione, are another important part of the non-enzymatic antioxidant system [3,44,45]. The increase in the content of low-molecular weight thiols (in particular glutathione) due to drought in wheat and maize reported earlier is proposed to counteract the induced oxidative stress [32,46]. Current results of low-molecular weight thiols are in line with these reports. The preliminary application of TAs tended to reduce the −SH content in plantlets subjected to water deficit, as compared to PEG-treated plants, which was better expressed in maize. Probably, the auxin compounds could regulate ROS homeostasis and decrease the oxidative stress caused by artificial drought. As a result, the necessity of maintenance of the cellular buffer capacity to high levels declined. Similar results and suggestions were reported for pea plants [16].

It is well known that CAT and POX are a key part of the enzymatic antioxidants responsible for the detoxification of ROS. The activities of CAT and POX were reported to increase due to drought stress in maize [46,47], which was also detected in our study. However, a decrease in these enzymatic activities was found in wheat. Vuković et al. [48] reported different response of CAT activity depending on the genotype-specific differences and on drought intensity. Authors suggested that the lack of CAT amplification boosted lipid peroxidation in the affected varieties. Therefore, the lack of induction in CAT and POX activities is considered to have a negative influence on ROS metabolism. Based on our results for growth inhibition in wheat due to PEG treatment, we also suppose the inability of CAT and POX to take part in detoxification of H_2_O_2_ overproduction. Shi et al. [44] showed that exogenous IAA decreased the contents of different ROS (H_2_O_2_ and O_2_^•−^) in Arabidopsis via induction of CAT, superoxide dismutase, glutathione reductase, and peroxidase activities. It was speculated that IAA have a major role in the control of drought stress response as auxins could modulate ROS generation and activate a cascade of other stress-responsive hormones [9]. The preliminary application of TAs before PEG treatment led to the adjustment of CAT and POX activities, and also suggested that the auxin compounds played an essential role in balancing H_2_O_2_ levels (production or decomposition).

Here, for the first time, we showed that the pre-application of the auxin-type PGR, TA-12 and TA-14, can modulate growth, content of the non-enzymatic antioxidants (proline, low-molecular weight thiols, phenolics) and activities of antioxidant enzymes (CAT and POX) in two economically important cereal crops subjected to PEG treatment. However, although maize is more susceptible to drought than wheat, it also showed adaptation to water stress due to TAs pre-application, due to the adjustment of non-enzymatic and enzymatic antioxidants close to physiological levels.

This study demonstrated that the preliminary application of TA-12 and TA-14 could improve the physiological responses of wheat and maize cultivars subjected to water deficit stress grown in Bulgaria. To confirm this prospective result more investigations are necessary, aiming to reveal in detail TAs modulating effect in plants grown under stress.

## 4. Materials and Methods

Seeds of winter wheat (*Triticum aestivum* L., cv. Sadovo-1) were obtained from the Institute of Plant Genetic Resources (Sadovo, Bulgaria) and maize (*Zea mays* L., cv. Kneja 307) were obtained from the Maize Research Institute (Knezha, Bulgaria). The plants were grown as water culture without aeration in a growth chamber (16/8 h photoperiod, 60–70% relative water humidity, 160 µmol m^−2^s^−1^ photon flux density, 24 ± 2 °C). Thirteen-day old plantlets were sprayed with 1mM solutions of TA-12 or TA-14 using Tween-80 (0.01%) as surfactant. The compounds are synthesized, characterized [49] and are available in the Nature Research Centre, Institute of Botany, Lithuanian Academy of Sciences, Vilnius, Lithuania. Twenty-four hours after the foliar treatment, part of the seedlings were subjected to water solution of polyethylene glycol 6000 (10% (−0.2 MPa)) imitating moderate drought stress in the nutrient solution [50,51].

Measurements of growth parameters (fresh weight and length of shoots and roots) were performed 72 h after PEG application using a ruler and electronic balance (Precision Standard TS4000, Ohaus^®^, Parsippany, NJ, USA). Samples for the biochemical analyses were collected from the aboveground part of plants and after weighing were immediately frozen in liquid nitrogen. Approximately 250 mg of leaf material was homogenized in 4 mL 1% trichloroacetic acid and then centrifuged (15,000× *g* for 30 min at 4 °C). The resulting supernatant was used for measuring the content of free proline, free low-molecular weight thiols, and total phenolics. The content of free proline was measured after acid derivatization with ninhydrin reagent on a water bath for 1 h [52]. The reaction was terminated by placing the samples in ice. The optical density was measured at 520 nm. The free low-molecular weight thiols were measured using aliquot of supernatant supplemented with Ellman’s reactive and after incubation at room temperature, the absorbance was measured at 412 nm [53]. The total phenolics content was measured according to Swain and Goldstein [54]. The supernatant was incubated with Folin–Ciocalteu reagent for 3 h at room temperature. The optical density was read at 725 nm. Gallic acid was used as a standard. For determination of the total protein content and the activities of catalase and guaiacol peroxidase, approximately 250 mg of leaf material was ground in cold isolating buffer (100 mM K_2_HPO_4_/KH_2_PO_4_, pH 7.0, with 1 mM EDTA) containing 1% polyvinylpyrolidone and centrifuged for 30 min at 15,000× *g* at 4 °C. The total soluble protein content was measured according to Bradford [55]. As a standard, bovine serum albumin was used. The activity of catalase (EC 1.11.1.6) was measured by monitoring the degradation of hydrogen peroxide at 240 nm [56]. The guaiacol peroxidase (EC 1.11.1.7) activity was determined by following the change of the absorbance at 470 nm according to the method of Dias and Costa [57]. As a donor of electrons, 1% guaiacol was used. The supernatants were obtained on a refrigerated Sigma 2-16K centrifuge (SciQuip, Wem, UK), and the spectrophotometric measurements were done on a Multiskan Spectrum spectrophotometer with microplate reader (Thermo Electron Corporation, Vantaa, Finland) and on Shimadzu UV-1601 spectrophotometer (Shimadzu, Kyoto, Japan).

The experiments were repeated three times in three replicates of each treatment. The data presented are mean values with ±SE. The significance of the different treatments was analyzed by one-way ANOVA with post-hoc Duncan’s multiple range test (*p* < 0.05).

## Data Availability

Not applicable.
